# Characteristics relating to the interiorization of acquired immunodeficiency syndrome in Brazil: a cross-sectional study

**DOI:** 10.1186/s40249-015-0060-2

**Published:** 2015-07-11

**Authors:** Gabriel de Deus Vieira, Ana Raquel Paz dos Reis, Francisco Ormidiel Teles de Alcântara Augusto, Karina Reis Martins, Paulo Roberto Fernandes Kern, Thairini Fuza de Souza, Sérgio de Almeida Basano, Luís Marcelo Aranha Camargo, Camila Maciel de Sousa

**Affiliations:** Departament of Medicine, São Lucas College, Porto Velho, Rondônia Brazil; Tropical Medicine Center of Rondônia, Porto Velho, Rondônia Brazil; Institute of Biomedical Sciences 5, University of São Paulo, Monte Negro, Rondônia Brazil

**Keywords:** Epidemiology, Aids, Residence characteristics, Amazon

## Abstract

**Background:**

In recent years there has been changes in the social and geographic profile of acquired immunodeficiency syndrome (AIDS). The aim of this study was to evaluate the internalization of AIDS in the state of Rondônia, Brazil.

**Findings:**

In Rondônia, 1473 AIDS cases were reported, with an average annual incidence of 15.8/100,000 persons (42.7 % women). The most common mode of viral transmission was sexual (96.5 %), and the majority of the individuals had not completed their primary education (64.8 %). There was heterogeneity in relation to case distribution, involving almost all of the municipalities in the state. The average annual mortality rate was 2.5/100,000 persons.

**Conclusion:**

Rondônia has a higher incidence of AIDS than the national average and the northern region. Efforts to provide access to treatment and follow-up of these individuals should be implemented, prioritizing areas where the incidence is higher and decentralizing the treatment of patients with AIDS in the state.

**Electronic supplementary material:**

The online version of this article (doi:10.1186/s40249-015-0060-2) contains supplementary material, which is available to authorized users.

## Multilingual abstracts

Please see Additional file 1 for translations of the abstract into the six official working languages of the United Nations.

## Introduction

Acquired immunodeficiency syndrome (AIDS) is considered a major public health problem. In recent decades, few diseases have generated as much interest from the scientific community and health professionals as AIDS, because of the social impact caused by the disease [1]. Currently, 1.7 million people are infected with the human immunodeficiency virus (HIV) in Latin America. In Brazil, approximately 34,500 new cases are documented annually [2].

With the advent of highly active antiretroviral therapy (HAART), the life expectancy of these individuals has increased significantly [3]; consequently, AIDS-related mortality has also decreased. This decrease in mortality began in 1996 with the provision of free HAART for HIV-infected individuals in Brazil. However, AIDS remains a serious and incurable disease in spite of the therapy, and, when untreated, can lead to death resulting from opportunistic diseases and comorbidities [4].

Currently, AIDS is an epidemic disease with a predominance of heterosexual transmission that affects the general population, regardless of gender or sexuality; it has ceased to be a disease that primarily affects the homosexual male population. A recent shift in the HIV-affected population has occurred, and it now also affects a considerable number of poor heterosexual women [5,6].

Other recent changes include the geographical distribution of the disease, with a move from large urban centers to the internal Brazilian states, termed interiorization, and the disease now affects individuals with low socioeconomic status and educational levels, termed pauperization. Brazil is a country with a high degree of social inequality, an uneven distribution of income, and limited education and health services in poorer areas, which contributes to this AIDS-related pauperization [6].

Recent studies in Rondônia show that there has been a recent increase in the number of HIV/AIDS cases [7, 8]. Thus, this study aimed to evaluate the epidemiological data and interiorization of AIDS in the state of Rondônia, Brazil.

## Methods

This is a descriptive ecology study using data from AIDS cases reported in the state of Rondônia during the period 2007–2012. Statistical data were provided by the State Agency of Health Surveillance of Rondônia (AGEVISA), through the Information System for Notifiable Diseases (SINAN). The variables included gender, age, ethnicity, education, mode of transmission, and outcome.

The incidence per 100,000 persons/year based on the absolute population residing in the state was calculated. Data analysis was performed using Microsoft Excel ® 2010 BioEstat 5.3 and GraphPad Prism version 5.0 software; the TabWin program (http://www2.datasus.gov.br/DATASUS/index.php?area=060805) was used to construct the map.

## Findings

**Fig. 1:**
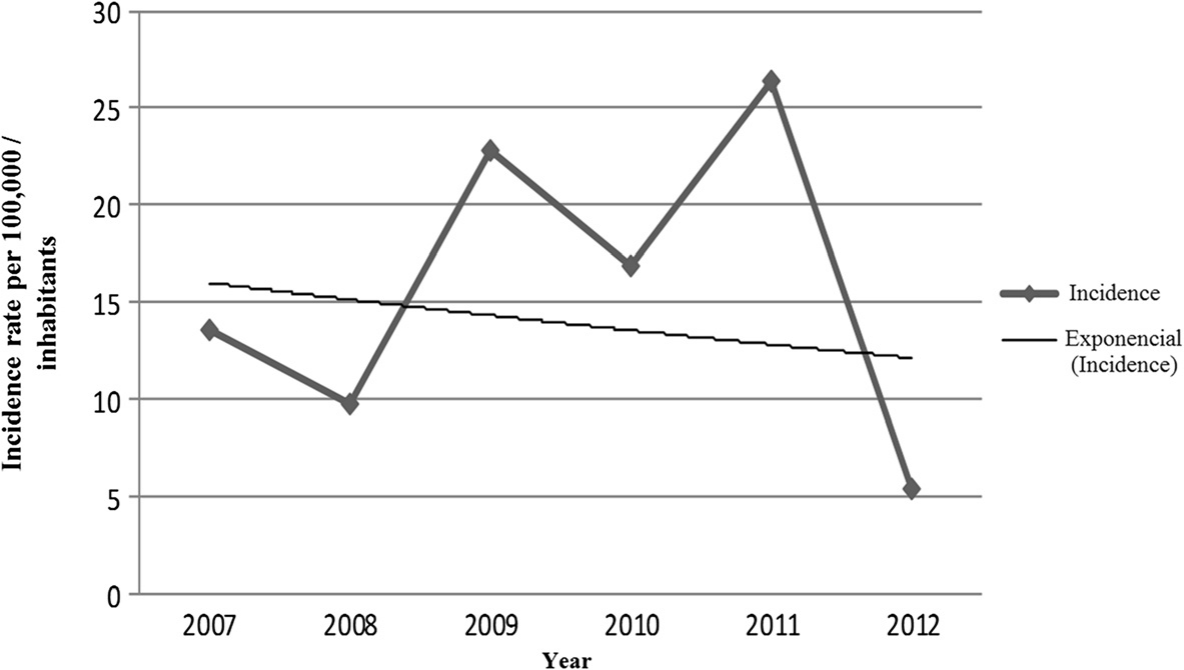
Annual incidence of diagnosed acquired immunodeficiency syndrome (AIDS) cases, per 100,000 persons, in the state of Rondônia, 2007–2012. Source: Information System for Notifiable Diseases SINANW and SINAN NET

During 2007–2012, 1473 cases of AIDS (13–81 years old) were reported in Rondônia, with an average annual incidence of 15.8/100,000 persons (Fig. 1). Of these, 843 (57.3 %) were men, with a mean age of 39 years, and 630 (42.7 %) were women, with a mean age of 36 years. The most prevalent age group was those aged 20–39 years (58.6 %). A mixed ethnicity was reported by 916 (64.1 %) individuals, and 814 (64.8 %) had not completed their elementary education. The most common mode of viral transmission was sexual (n = 1409, 96.5 %) (Table 1).

**Table 1:** Social and clinical data of patients with acquired immunodeficiency syndrome (AIDS) in Rondônia, 2007–2012

Variables	n	%	Unknown*
**Age range** (**years**)			42
13–19	31	2.2	
20–39	839	58.6	
40–59	503	35.1	
≥60	58	4.1	
**Ethnicity**			43
Mixed	916	64.1	
White	380	26.6	
Black	95	6.6	
Yellow	33	2.3	
Indigenous	6	0.4	
**Schooling**			216
Illiterate	73	5.8	
Incomplete primary	814	64.8	
Complete primary	105	8.4	
Incomplete high school	77	6.1	
Complete high school	108	8.6	
Incomplete college	35	2.7	
Complete college	45	3.6	
**Most likely mode of transmission**			14
Sexual contact with men	715	49	
Sexual contact with women	637	43.6	
Sexual contact with men and women	57	3.9	
Use of injectable drugs	42	2.8	
Blood transfusion	5	0.4	
Vertical transmission	2	0.2	
Hemophilia treatment	1	0.1	

*Lack of data, due to an incomplete notification form.

Source: Information System for Notifiable Diseases SINANW and SINAN NET

**Fig. 2:**
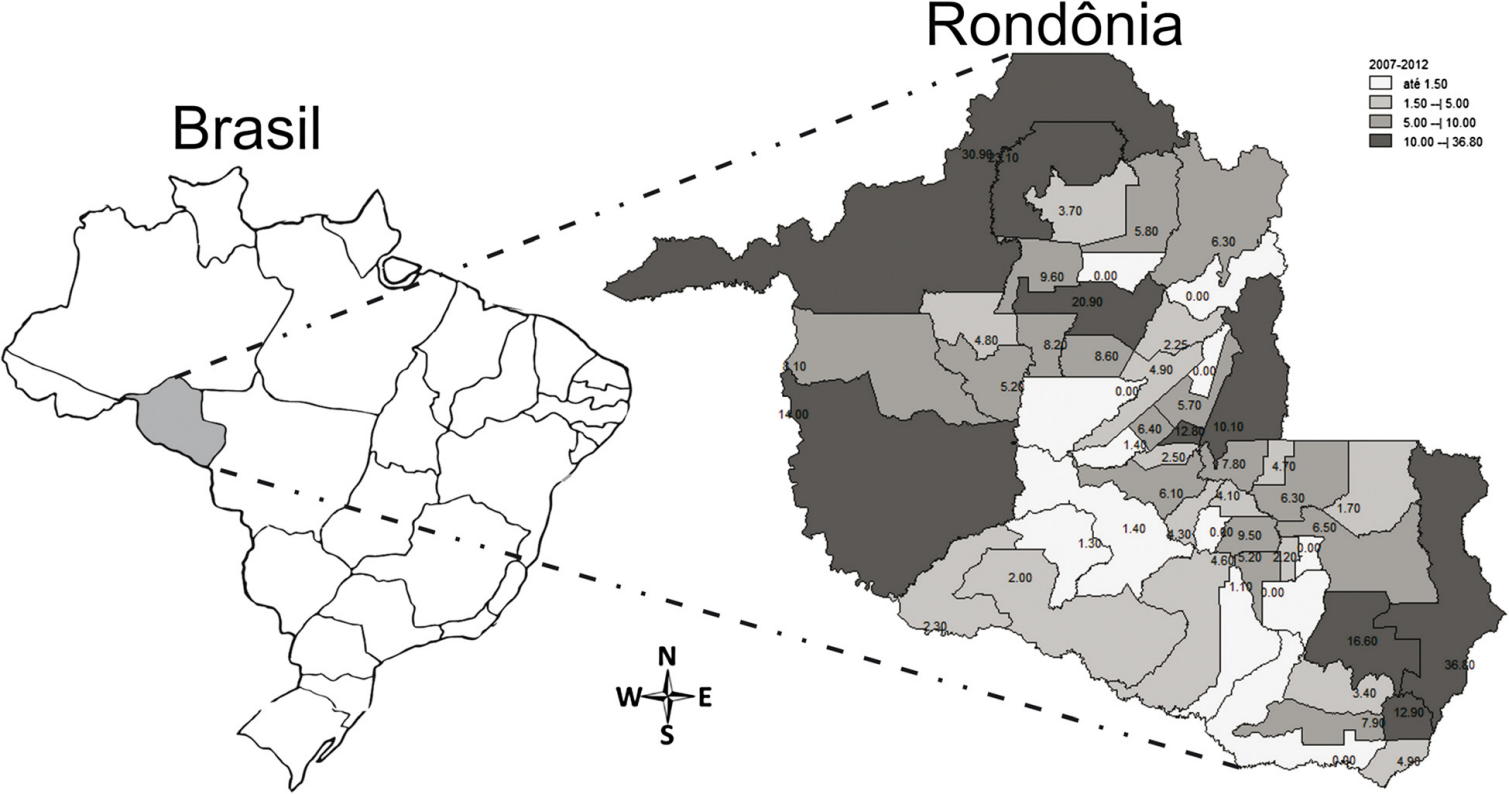
Annual incidence of diagnosed acquired immunodeficiency syndrome (AIDS) cases, per 100,000 persons, by municipality in the state of Rondônia, 2007–2012. Source: Information System for Notifiable Diseases SINANW and SINAN NET

The municipalities with the highest incidence rates were Vilhena (36.8/100,000 persons/year), Porto Velho (30.9/100,000 persons/year), Candeias do Jamari (23.1/100,000 persons/year), and Ariquemes (20.9/100,000 persons/year) (Fig. 2). Regarding the progression of the cases, 232 (15.7 %) patients died, with an average annual mortality of 2.5/100,000 persons (Table 2).

**Table 2:** Data related to the prevalence and deaths of acquired immunodeficiency syndrome (AIDS) cases in the state of Rondônia, 2007–2012

Year	Variables					
	n	*fr*	Deaths (n)	Population	Mortality rate*	% Deaths
**2007**	217	0.1473	54	1,590,027	3.39	24.8
**2008**	146	0.0991	22	1,493,566	1.47	15.1
**2009**	343	0.2328	45	1,503,911	2.99	13.1
**2010**	264	0.1792	36	1,562,409	2.3	13.6
**2011**	417	0.2830	60	1,576,455	3.81	14.4
**2012**	86	0.0583	15	1,590,011	0.94	17.4
**TOTAL**	**1**,**473**		**232**			**15.7**

*Calculation based on 100,000 persons.

Source: Information System for Notifiable Diseases SINANW and SINAN NET

## Discussion

According to our results, the average incidence of AIDS in Brazil in the period 2007–2011 was 12.7 cases per 100,000 persons/year, while the average incidence in the northern region during the same period was 11.3 cases per 100,000 persons/year [9]. In that same study, it was reported that Rondônia had a higher incidence (15.8/100,000 persons/year) than the incidence that we report here.

In the present study, the proportion of each gender that was affected was fairly similar. At the beginning of the epidemic, men were primarily affected; however, AIDS cases have gradually appeared in the female population as a result of unprotected sexual intercourse, contributing to the “feminization” of the disease [10] and resulting in HIV/AIDS prevention public policies that target women beginning in the 1990s [11]. According to Rodrigues-Júnior and Castilho [12], the feminization of AIDS is related to the greater vulnerability of women compared to men, with difficulties in negotiating for protection during intercourse. It was noted in this study that most men and women are sexually active, thereby increasing the vulnerability of women, owing to their partners’ extramarital affairs [13]. This phenomenon has been described in other regions of Brazil [14].

Simon *et al.* [15], in their study of HIV in southern Brazil, reported that HIV was more commonly transmitted via sexual intercourse (86.3 %), with 20.5 % of the respondents involved in bisexual relations, followed by syringe use (8.8 %) and blood transfusions (2.5 %). In the present study, the most common mode of transmission was also sexual, of which 3.9 % of the respondents were involved in bisexual relationships; the most common age group was those aged 20–39 years. Therefore, the main route of transmission is currently sexual, primarily affecting an age group that tends to be more sexually active [11].

The interiorization of the disease has also been described previously, as a phenomenon that complicates the treatment and monitoring of these individuals owing to the distance from large urban centers, leaving the HIV-infected residents of these areas more vulnerable to the disease [16, 17]. Stephan *et al.* [18], in their study of the spatial distribution of AIDS in Campinas-SP, concluded that there was a higher concentration of the disease in the areas with the worst living conditions. The disease also tends to affect individuals with lower levels of education, contributing to the pauperization of AIDS [10, 19].

With the advent of antiretroviral therapy, there has been a significant reduction in disease-related mortality. However, the mortality rate in Rondônia remains high at 15.7 %; this may be related to delayed diagnosis, difficulties in treatment adherence, and ineffective diagnosis and treatment of comorbidities [20].

This study has certain limitations, including the amount of missing information in the database and lack of reported cases in the state, contributing to the underreporting of the disease. The information systems are important epidemiological tools that provide knowledge of the health situation, information regarding the status of a specific disease in a given region, and direction for public policy to address the priority needs of the population. However, in the present case, the real impact of the disease may not be well understood given the possibility of underreporting [21].

## Conclusion

There has been a recent migratory influx of thousands of male workers to the state of Rondônia; the majority of these men are poorly educated with low incomes, which impacts the already weak state public health system, negatively influences various social indices, and may contribute to the increase in HIV-infected people in addition to the ineffectiveness of preventive measures for HIV transmission, owing to the increased prostitution, alcoholism, and illicit drug use observed with this influx of migrants. Currently, Rondônia has a higher incidence of this disease when compared to the national and northern region averages, reflecting the interiorization, pauperization, and feminization of the disease that has been observed in the rest of Brazil, making women extremely vulnerable to this disease. Efforts to improve treatment and follow-up of these individuals are being conducted with the decentralization of the specialized care services by the state and the increased capacity of the service network. However, health education initiatives should continue; as the epidemic advances to the inland areas of the state and affects a population that has been neglected by public authorities, the impact and consequences of the disease may decline not only for the transmission of the virus but also for disease-related morbidity and mortality.

## Additional file

Additional file 1:
**Multilingual abstracts in the six official working languages of the United Nations.**

## Abbreviations

HIV: Human Immunodeficiency Virus
AIDS: Acquired Immunodeficiency Syndrome
HAART: Highly Active Antiretroviral Therapy
AGEVISA: State Agency of Health Surveillance of Rondônia
SINAN: System for Notifiable Diseases
