# Supplementary material for: Characteristics relating to the interiorization of acquired immunodeficiency syndrome in Brazil: a cross-sectional study
Source: Infect Dis Poverty. 2015 Jul 11;4:31. doi: 10.1186/s40249-015-0060-2 (PMC4539930; doi:10.1186/s40249-015-0060-2)
Supplement: Additional file 1: — Multilingual abstracts in the six official working languages of the United Nations. [file 40249_2015_60_MOESM1_ESM.pdf]

Translation of the abstract into the six official working languages of the United Nations

الخصائص المرتبطة باستبطان الإيدز في البرازيل: دراسة عرضية

جابرييل دي ديوز فييرا، أنا راكويل باز دوز ريز، فرانسيسكو أورميديال تيليز دي ألكانترا أوجستو، كارينا ريز مارتنز، باولو روبرتو فيرنانديز كيرن، ثايريني فوزا دي سوزا، سيرجيو دي ألميدا باسانو، لويس مارسيلو أرانها كامارجو، كاميلا ماسيل دي سوزا.

الملخص

الهدف: كان الهدف من هذه الدراسة هو تقييم استبطان متلازمة نقص المناعة المكتسب (الإيدز) في روندونيا، البرازيل.

طرق العمل: تم إجراء دراسة وبائية وصفية لحالات الإيدز المسجلة بين عامي 2007 و 2012 في نظام معلومات الأمراض السارية. وتم دراسة الخصائص الاجتماعية والطبية للحالات، بالإضافة إلى التوزيع المكاني للمرض في الولاية.

النتائج: في روندونيا، تم الإبلاغ عن 1473 حالة إصابة بالإيدز بين عامي 2007 و 2012 ، بمتوسط معدل سنوي15.8/ 100.000 شخص 15.8/100,000 شخص (42.8 % من النساء). كانت أكثر وسيلة شيوعاً لانتقال الفيروس هي الجنس (95.5 %)، ولم يكمل غالبية الأفراد تعليمهم الابتدائي (64.8 %). كان هناك تباين فيما يتعلق بتوزيع حالات الإصابة، تشمل تقريباً جميع البلديات في الولاية. وكان متوسط معدل الوفيات السنوي 2.5 /100.000 شخص.

الاستنتاج: روندونيا لديها معدل أعلى للإصابة بالإيدز من المتوسط الوطني ومن متوسط الإصابة بالمنطقة الشمالية. ينبغي تنفيذ الجهود الرامية إلى توفير فرص الحصول على العلاج ومتابعة حالات الإصابة بالإيدز، وإعطاء الأولوية للمناطق ذات المعدل الأعلى من الإصابة وتحقيق اللامركزية في علاج مرضى الإيدز بالولاية.

Translated from English version into Arabic by Saher Salama, through

TRANSLATORS
WITHOUT BORDERS

巴西艾滋病内在流行特征的横断面研究

加布里 埃尔神 维埃拉，安娜 斯雷斯，弗朗西斯科 奥古斯托，卡琳娜 马丁斯，圣保罗罗伯托 斯克恩，德索萨，塞尔吉 奥巴萨农，马塞洛卡马乔，卡米拉 德索萨

摘要

目的：评估巴西朗多尼亚州艾滋病内在的流行特征。

方法：本研究对 2007-2012 年法定传染病信息系统中的艾滋病病例进行了描述性流行病学研究，分析了这些病例的社会、临床特点，以及空间分布特征。

结果：2007-2012 年，朗多尼亚州有 1,473 例艾滋病病例报告，年平均发病率为 15.8/100,000 人，其中 42.8%为女性。最常见的病毒传播方式是性传播（96.5%），患者大多未完成小学教育（64.8%）。该州近乎所有城市的病例分布不均衡，年平均死亡率为 2.5/100,000 人。

结论：朗多尼亚州的艾滋病发病率不仅高于全国平均值，也高于北部地区。因此，该州必须尽力给艾滋病患者提供诊治机会，优先治疗高发病区患者，简化就诊程序。

Translated from English version into Chinese by Chen Jin, edited by Yang Pin, through

CDC CHINESE CENTER FOR DISEASE CONTROL AND PREVENTION
NATIONAL INSTITUTE OF PARASITIC DISEASES

Caractéristiques liées à l'intériorisation du SIDA au Brésil : étude transversale

Gabriel de Deus Vieira, Ana Raquel Paz dos Reis, Francisco Ormidiel Teles de Alcântara Augusto, Karina Reis Martins, Paulo Roberto Fernandes Kern, Thairini Fuza de Souza, Sérgio de Almeida Basano, Luís Marcelo Aranha Camargo, Camila Maciel de Sousa

Résumé

Objectif : Le but de notre étude était d'évaluer l'intériorisation du syndrome d'immunodéficience acquise (SIDA) dans l'état de Rondônia au Brésil.

Méthodes : Nous avons mené une étude épidémiologique descriptive des cas de SIDA enregistrés entre 2007 et 2012 dans le Système d'information sur les maladies à déclaration obligatoire. Les caractéristiques sociales et cliniques des cas ainsi que leur répartition géographique dans l'état de Rondônia ont été étudiées.

Résultats : Entre 2007 et 2012, 1473 cas de SIDA ont été rapportés dans l'état de Rondônia, soit une incidence annuelle moyenne de 15,8 cas pour 100 000 habitants (42,8 % de femmes). La transmission du virus s'est effectuée la plupart du temps par voie sexuelle (96,5 %) et la majorité des personnes affectées n'avait pas dépassé le niveau de scolarité primaire (64,8 %). La distribution des cas était hétérogène et couvrait presque toutes les municipalités de l'état. Le taux de mortalité annuelle moyen était de 2,5 pour 100 000.

Conclusion : L'état de Rondônia présente une incidence du SIDA plus élevée que la moyenne nationale et que le Nord du Brésil pris dans son ensemble. Des efforts doivent être entrepris pour faciliter l'accès au traitement et suivre les patients atteints du SIDA, en visant en priorité les zones de forte incidence et en décentralisant le traitement des patients à l'échelle de l'état.

Translated from English version into French by Suzanne Assenat, through

TRANSLATORS
WITHOUT BORDERS

Характеристики, связанные с интериоризацией СПИДа в Бразилии: кросс-секционное исследование

Габриэль де Деус Виейра, Ана Ракель Пас дос Рейс, Франциско Ормидель Телес де Алькантара Августо, Карина Рейс Мартинс, Пауло Роберто Фернандес Керн, Таирини Фуза де Суза, Серхио де Алмеида Басано, Луис Марсело Аранха Камарго, Камила Масиэль де Суса

Краткое описание

Цель: Данное исследование было направлено на оценку интериоризации синдрома приобретенного иммунодефицита (СПИДа) в штате Рондония, Бразилия.

Методология: Было проведено описательное эпидемиологическое исследование случаев заражения СПИДом, зарегистрированных в период с 2007 по 2012 год в Информационной системе заболеваний, подлежащих обязательной регистрации. Были изучены социальные и клинические характеристики случаев, в также пространственное распространение вируса в штате.

Результаты: В Рондонии в период с 2007 по 2012 годы было зарегистрировано 1473 случая заражения СПИДом при средней частоте возникновения заболевания, равной 15,8/100 000 человек (из них 42,8% женщин). Наиболее распространенным способом передачи вируса был половой путь (96,5%), при этом большинство заболевших не получили даже начального школьного образования (64,8%). Кроме того, наблюдалась неоднородность в отношении распространения случаев заражения, включая почти все муниципалитеты штата. Средний годовой уровень смертности составил 2,5/100 000 человек.

Выводы: В Рондонии зафиксирована более высокая частота возникновения СПИДа, чем в среднем по стране и в северном регионе. Необходимо принять меры по обеспечению доступа к медицинскому обслуживанию и последующему отслеживанию случаев заражения СПИДом, обращая особое внимание на зоны с более высокой частотой возникновения заболевания и децентрализацию оказания медицинских услуг больным СПИДом на территории штата.

Translated from English version into Russian by Irina Zayonchkovskaya, through

TRANSLATORS WITHOUT BORDERS

Características de la interiorización del SIDA en Brasil: un estudio transversal

Gabriel de Deus Vieira, Ana Raquel Paz dos Reis, Francisco Ormidiel Teles de Alcântara Augusto, Karina Reis Martins, Paulo Roberto Fernandes Kern, Thairini Fuza de Souza, Sérgio de Almeida Basano, Luís Marcelo Aranha Camargo, Camila Maciel de Sousa

Resumen

Objetivo: El propósito del presente estudio es evaluar la interiorización del síndrome de inmunodeficiencia adquirida (SIDA) en Rondônia, Brasil.

Métodos: Se llevó a cabo un estudio epidemiológico descriptivo de casos de SIDA registrados en el Sistema de Información para Enfermedades Reportables entre los años 2007 y 2012. Se estudiaron las características sociales y clínicas de los casos así como también la distribución espacial de la enfermedad en el estado.

Resultados: En Rondônia, se reportaron 1.473 casos de SIDA entre los años 2007 y 2012, con una incidencia anual promedio de 15,8/100.000 personas (42,8% mujeres). La forma más común de transmisión viral fue la sexual (96,5%), y la mayoría de las personas no habían completado su escuela primaria (64,8%). Hubo heterogeneidad en cuanto a la distribución de los casos, estando involucradas prácticamente todas las municipalidades del estado. La tasa de mortalidad anual promedio fue de 2,5/100.000 personas.

Conclusión: Rondônia tiene una incidencia de SIDA más alta que el promedio nacional y que el de la región norte. Deberían implementarse esfuerzos para ofrecer acceso a tratamiento y seguimiento para casos de SIDA, dando prioridad a las zonas donde la incidencia es más alta, y descentralizando el tratamiento de los pacientes con SIDA en el estado.

Translated from English version into Spanish by Maria Alejandra Aguada, through

TRANSLATORS WITHOUT BORDERS
